# Impacts of Morphology on the Fracture Resistance of the High-Strength Dual-Phase Steels

**DOI:** 10.3390/ma18102253

**Published:** 2025-05-13

**Authors:** Hao Xu, Zhihong Jia, Qingquan Lai

**Affiliations:** 1 Key Laboratory for Light-Weight Materials, Nanjing Tech University, Nanjing 211816, China; 202261203256@njtech.edu.cn; 2 Materials Academy, Jiangsu Industrial Technology Research Institute, Suzhou 215131, China

**Keywords:** dual-phase steels, morphology, fracture toughness, damage, plasticity

## Abstract

A good combination of strength and fracture resistance is highly desired for the development of high-strength ferrite–martensite dual-phase (DP) steels for automotive application. But the increase in strength is usually compromised by a reduction in fracture resistance, and the guideline for microstructure optimization remains to be established. This study is dedicated to the DP steels with tensile strength above 1 GPa, and the influences of the equiaxed and fibrous morphologies on the mechanical properties were investigated by both the uniaxial tensile tests and the essential work of fracture (EWF) method. The fibrous morphology is efficient in increasing strength due to the ferrite grain refinement effect. Under uniaxial tension, the fibrous DP morphology does not lead to higher fracture strain. But when evaluating with the EWF method, the fibrous DP steels present a superior fracture resistance, which is attributed to the larger crack tip necking. The interpretation of the fracture resistance measurements was substantiated by the detailed damage observations. Therefore, the fibrous DP concept could provide an efficient pathway to improve the combination of strength and fracture resistance.

## 1. Introduction

The increasingly stringent demands in passenger safety and weight reduction in the automotive industry constitutes a main driving force for the development of advanced high-strength steels (AHSSs) [1,2,3]. Ferrite–martensite dual-phase (DP) steel is one of the major AHSSs widely used in the car body-in-white for the good combination of strength and ductility, as well as the low cost [4]. However, although with a good ductility, the DP steels were frequently found in practice to suffer from a lack of local formability, e.g., the problem of edge cracking during the forming operations of the pre-cut steel sheets. The lack of local formability is related to the insufficient fracture toughness, and the key is to improve the performance of DP steels in resisting the propagation of the pre-existing defects [5,6].

The DP steels involve the composite microstructure consisting of the strong martensite islands embedding in the soft ferrite matrix. The yielding of DP steels is controlled by the yielding of the soft ferrite, and the plastic incompatibility between ferrite and martensite results in the enhanced generation of geometrically necessary dislocations and a distribution of internal stresses to increase the strain hardening rate [7]. However, the increased local stress level could lead to the occurrence of damage events, either by the decohesion of ferrite/martensite interface or by the cracking of martensite islands. It has been summarized in a recent report [8] that the dominant damage mechanism primarily depends on the martensite volume fraction. In the DP steels with a low martensite volume fraction, the major damage mechanism is interface decohesion, while for DP steels with increased martensite volume fraction, martensite cracking is dominant. The ductile fracture of DP steels is proceeded by the nucleation, growth, and coalescence of voids [9]. It could be envisaged that the approaches of improving the fracture resistance of DP steels rely on the control of the damage accumulation processes, including delaying the nucleation or coalescence of voids.

Extensive efforts have been made to improve the fracture resistance of DP steels. A large part of these investigations are related to tailoring the strength contrast between ferrite and martensite. On one hand, the strength contrast between ferrite and martensite could be reduced by decreasing the strength of martensite, either achieved by conducting the tempering treatment [10,11] or by designing low carbon content in martensite [12]. The delay of void formation has been evidenced, but the improvement in fracture resistance is obviously compromised by a decrease in strength. On the other hand, the ferrite matrix could be strengthened by the precipitation of nano-sized vanadium carbide [13,14] or by substantial grain refinement [15,16,17]. Simultaneous enhancement of strength and fracture resistance could be achieved by this approach, but the sophisticated thermomechanical processing and the associated cost have set the challenges for industrial implementation. A noticeable recent advance is the re-examination of the concept of fibrous DP steels, also called “Thomas fibers” DP steels [18]. It is shown that simply the change in morphology from the classical equiaxed to the fibrous one could allow a significant increase in fracture resistance [19,20,21]. However, the effectiveness of the fibrous DP concept has only been studied with steel of a grade below 800 MPa. The examination of the fibrous DP concept should be extended to higher strength level, especially when one considers the significant impacts of strength on the damage accumulation [22,23].

This work is dedicated to investigating the impacts of morphology on the fracture resistance of ferrite–martensite dual-phase steels with strength beyond 1 GPa. Systematic comparison was made between microstructures with the equiaxed morphology, which is typical for conventional cold-rolled DP steels, and with the fibrous morphology. Considerations have been made on the assessment of fracture properties, because the ranking of materials depends on the protocol and specimen configuration of the mechanical testing [24,25,26]. The fracture resistance was assessed by both the uniaxial tension and the fracture toughness tests, involving different levels of stress triaxiality. The essential work of fracture (EWF) method was used to evaluate the fracture toughness of the steel sheets [27,28]. High-resolution X-ray computer tomography (CT) has been used to characterize and quantify the damage evolution. A combination of the mechanical and microstructural characterizations could provide a deeper insight into the facture mechanism and could identify the controlling microstructural features for fracture resistance, formulating the guideline for optimizing the high-strength DP steels with improved mechanical performance.

## 2. Materials and Methods

**Figure 1:**
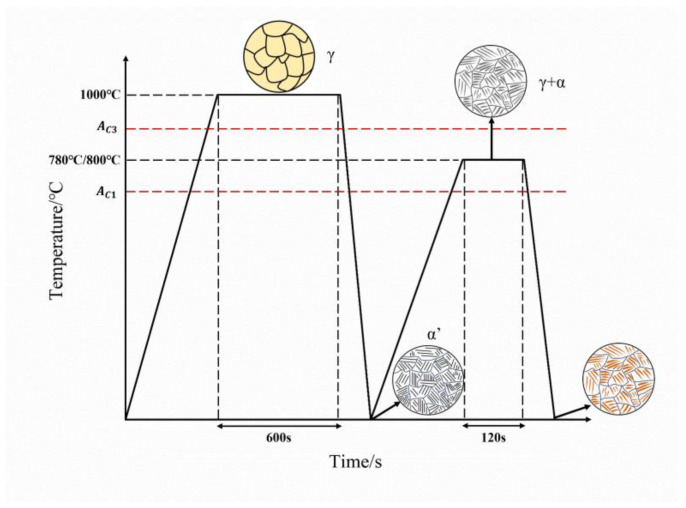
Schematic illustration of the processing route to produce fibrous DP steels.

A commercial dual-phase steel DP980 with the thickness (*t*_0_) of 1.4 mm was used in this study, with the chemical composition of Fe-0.09C-0.3Si-2.25Mn. The heat treatment to process the fibrous DP steels was performed on the Thermecmastor thermomechanical simulator. The as-received material was heated to 1000 °C, held for 10 min, and quenched to room temperature. Intercritical annealing was subsequently performed at 780 °C and 800 °C for 2 min. The schematic illustration of the processing route is shown in Figure 1.

Microstructures were observed on the plane normal to the transverse direction. The samples were cut, ground, polished, and etched by 2% Nital solution. The microstructures were characterized by means of optical microscope (OM) and scanning electron microscope (SEM). The volume fraction of martensite was measured on the SEM micrographs with the ImageJ software (V1.8.0.112).

**Figure 2:**
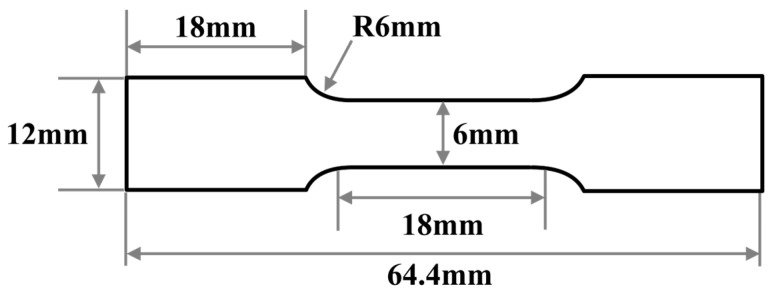
Design and dimension of the tensile specimens.

The mechanical properties were firstly evaluated by uniaxial tensile testing. Three tests were conducted on each microstructure. The dog-bone-shaped tensile specimens were prepared by electrical discharge machining (EDM) with tensile direction parallel to the rolling direction, with the length and width of the gauge section being 20 mm and 6 mm, respectively. The design and dimension of the tensile specimen is shown in Figure 2. The uniaxial tensile tests were carried out at a strain rate of 10^−3^/s, and a contact extensometer was used for the strain measurement. The fracture stress was calculated by dividing the load at fracture by the fracture surface area (*A_c_*). The fracture strain (*ε_f_*) was determined by the following equation [29]:

(1) εf=lnA0Ac,

where *A*_0_ is the initial cross-section area.

The fracture resistance was further evaluated by the EWF method, which was developed for the ductile thin sheets [30,31]. The double-edge-notched tension (DENT) specimens were prepared by EDM and the notch was sharpened by a razor blade. The initial ligament length (*l*_0_) of the DENT specimens ranges from 4 to 6.5 mm. The integration of the load–displacement response provides the total work of fracture (*W_f_*). The specific fracture work (*w_f_*) can be calculated as:

(2) $$w_{f}=\frac{W_{f}}{l_{0}t_{0}}=w_{e}+\beta l_{0}w_{p},$$

where *w_e_* is the specific essential work of fracture, *w_p_* is the specific diffuse plastic work, and *β* is the shape factor of the diffuse plastic zone. A linear regression on *w_f_* as a function of *l_0_* allows the separation of *w_e_* and *βw_p_*. The *w_e_* is the fracture toughness parameter to assess the resistance to crack propagation [19,24,32]. The equivalent fracture strain [19], $\varepsilon_{f}^{eq}$, of the DENT specimens is expressed as:

(3) $$\varepsilon_{f}^{eq}=\frac{2}{\sqrt{3}}ln\frac{t_{0}}{t_{f}},$$

where *t_f_* is the final thickness of the ligament of the broken DENT specimens.

The Zeiss 620 (Zeiss, Oberkochen, Germany) X-ray computer tomography (CT) was employed for the three-dimensional damage analysis. The X-ray scanning was operated at the voltage of 140 kV, and the current intensity was 150 μA. The exposure time was set as 2 s. The scanner was rotated by 180° to collect 2000 projection images, with the scanning resolution of 1.3 μm. The reconstruction of the data was performed by the Reconstructor Scout-and-Scan software (V2.5.1), and the Avizo software (Avizo 3D 2024.2) was used to create a three-dimensional visualization model. The pixel size was set as 1.3 μm × 1.3 μm × 1.3 μm. The data were segmented by the gray value threshold to extract the voids, and data with the objective smaller than 3 pixels were removed. To evaluate the damage evolution with strain, the voids at different locations of the necking zone were quantified, and the local thickness strain (*ε_t_*) was defined by the following equation [33]:

(4) $$\varepsilon_{t}=\ln\frac{t_{0}}{t},$$

where *t* is the local thickness of each slice.

## 3. Results

**Figure 3:**
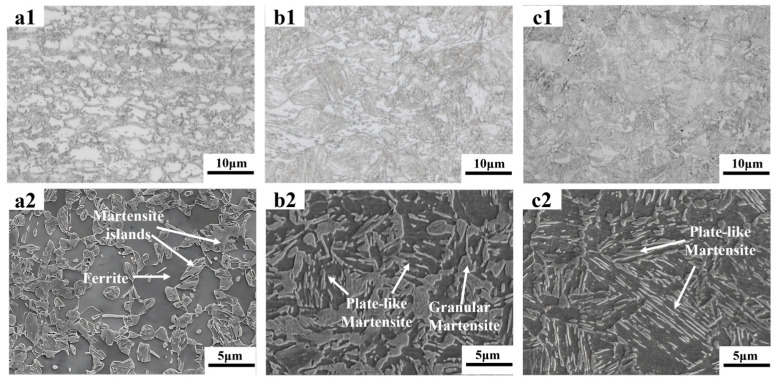
Microstructures of the DP steels: (**a1**,**a2**) are the OM and SEM micrographs of Equiaxed-49%; (**b1**,**b2**) are the OM and SEM micrographs of Fibrous-45%; (**c1**,**c2**) are the OM and SEM micrographs of Fibrous-26%, respectively.

Figure 3 shows the microstructures of DP steels with different morphologies. Figure 3(a1,a2) presents the microstructure of the as-received DP steels, which involves the equiaxed morphology with 49 vol.% martensite (Equiaxed-49%). The martensite islands are formed from the austenite that nucleates at the carbide particles at the grain boundaries of the equiaxed ferrite. Figure 3(b1,b2,c1,c2) are the DP microstructures processed through the route explained in Figure 1. Two kinds of martensite islands are found in such microstructures, including the granular one formed at the prior austenite grain boundaries and the plate-like one transformed from the martensite laths. It is the aggregate of these plate-like martensite islands that constitutes the fibrous morphology. The volume fractions of martensite in Figure 3(b1,b2) and Figure 3(c1,c2) are 45 vol.% (Fibrous-45%) and 26 vol.% (Fibrous-26%), respectively.

**Figure 4:**
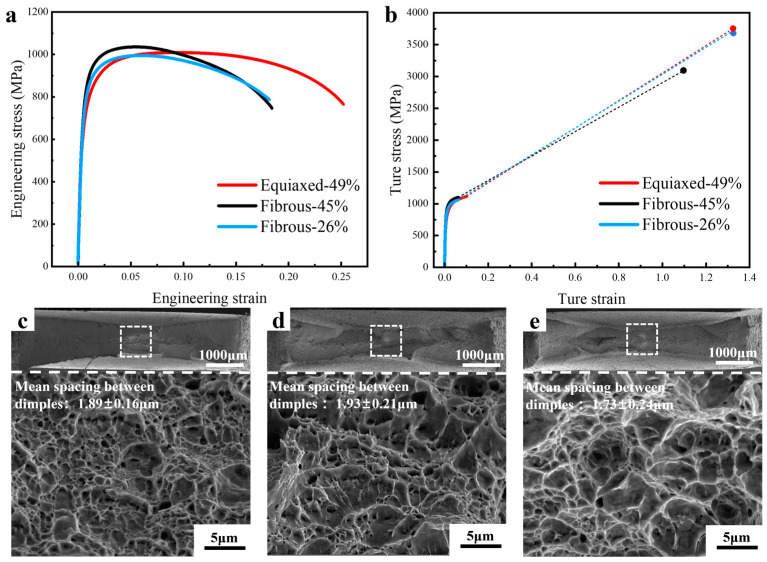
Tensile response and fracture surface analysis: (**a**) is the engineering stress–strain curves; (**b**) is the true stress–strain curves associated with the extension to the fracture stress and fracture strain with the dotted lines; (**c**–**e**) are overview and high-magnitude SEM micrographs of the fracture surfaces of Equiaxed-49%, Fibrous-45%, and Fibrous-26%, respectively, and the mean spacing between dimples are indicated.

The mechanical properties of the DP microstructures were firstly assessed by uniaxial tensile testing. Figure 4a shows that the Equiaxed-49% presents the lowest yield strength, but the strain hardening capability allows it to reach the tensile strength of 1 GPa and the best uniform elongation. Both fibrous DPs present a higher yield strength, but the strain hardening capability is weaker, resulting in an earlier onset of necking. The tensile strength of Fibrous-45% and Fibrous-26% are 1055 MPa and 1004 MPa, respectively. Therefore, the fibrous DP concept is effective in increasing strength, especially when one considers the lower martensite volume fraction of Fibrous-26%. All the microstructures fail in the ductile mode, with dimples covering the fracture surface of the broken specimens. The Equiaxed-49% shows a comparable fracture strain with the Fibrous-26%, and the Fibours-45% involves a lower fracture strain. The mean spacing between dimple centers is similar among these DP microstructures. The tensile properties are summarized in Table 1.

**Figure 5:**
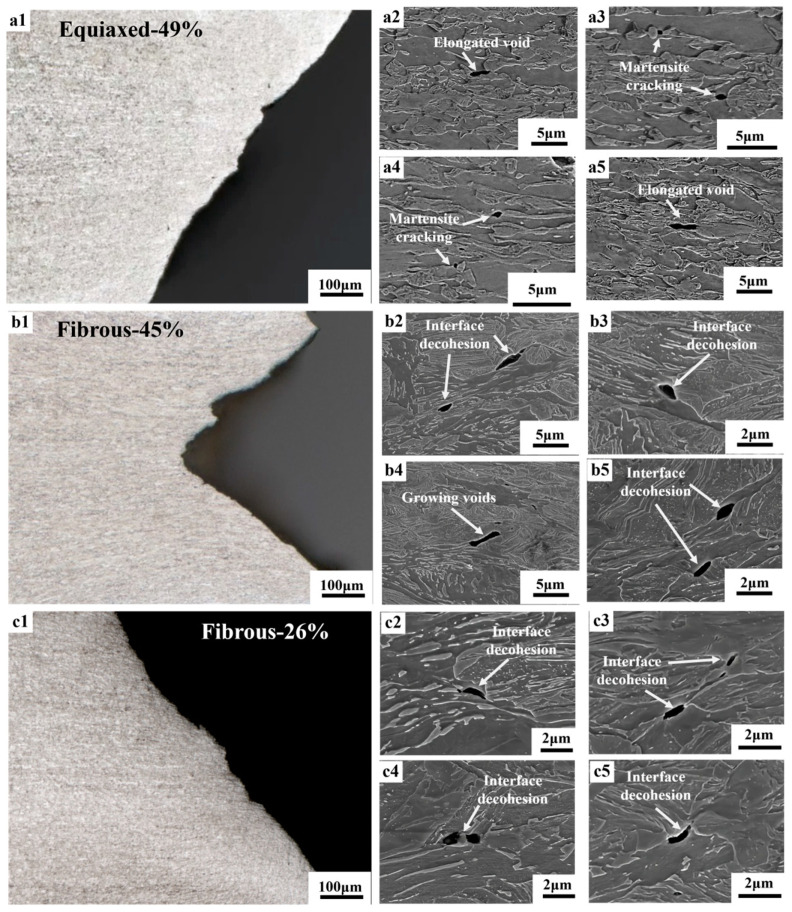
Damage observation of the DP microstructures: (**a1**–**a5**) are the OM and SEM micrographs of Equiaxed-49%; (**b1**–**b5**) are the OM and SEM micrographs of Fibrous-45%; (**c1**–**c5**) are the OM and SEM micrographs of Fibrous-26%.

**Figure 6:**
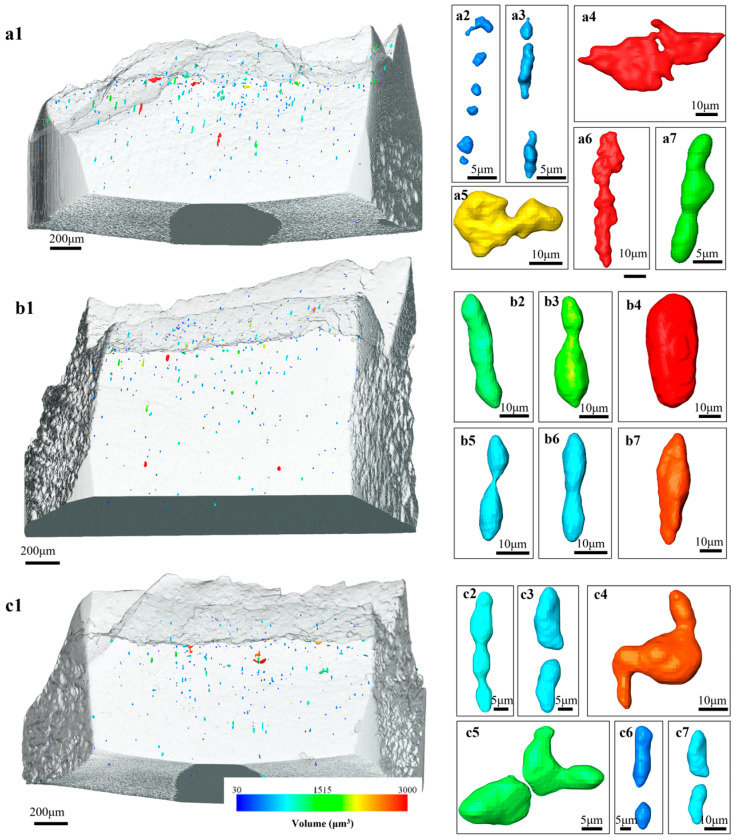
Damage observations by using the X-ray CT: (**a1**,**b1**,**c1**) show the overall distribution of voids in the broken tensile specimens of Equiaxed-49%, Fibrous-45%, and Fibrous-26%, respectively; (**a2**–**a7**) are the typical voids in Equiaxed-49%; (**b2**–**b7**) are the typical voids in Fibrous-45%; (**c2**–**c7**) are the typical voids in Fibrous-26%.

Figure 5 is the observations of the damage events adjacent to the fracture surface of the broken tensile specimens. In the Equiaxed-49% sample, voids are mainly formed by martensite cracking, and the voids can grow into the elongated shape along the tensile direction. For the fibrous DP microstructures, the majority of the voids are found to form at the ferrite–martensite interface. The initial penny-shape voids, after the interface decohesion, also evolve into the elongated voids, accompanying the severe plastic flow as indicated by the change in morphology. A necklace of voids along the tensile direction could be occasionally observed. Thereafter, X-ray CT was performed to characterize the three-dimensional distribution of voids and to obtain a statistical analysis. According to Figure 6, the density of voids is obviously increased with increasing strain in the necking zone. Being consistent with the SEM observations, the voids are found to grow more extensively along the tensile direction, evolving into the elongated shape. In addition, the distribution of voids is often found to align with the tensile direction, resulting in the preferred linkage of voids and in the formation of void necklace [34].

**Figure 7:**
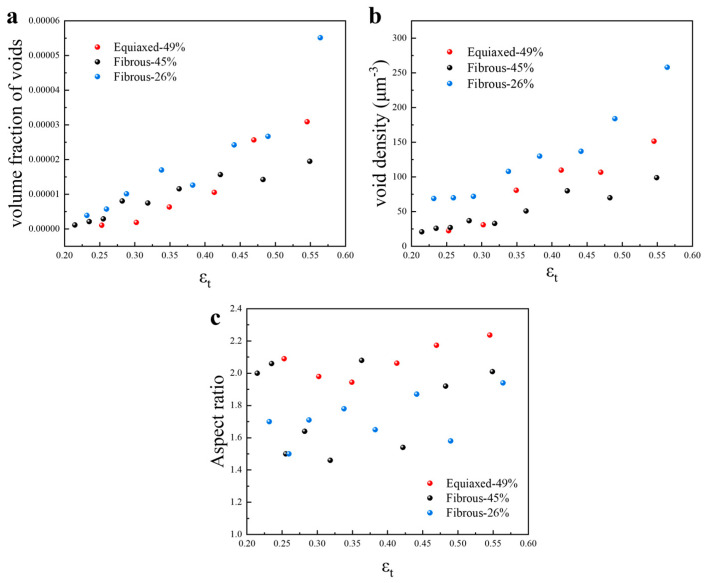
Quantification of the damage evolution in the tensile testing: (**a**) is the evolution of void volume fraction; (**b**) is the evolution of the void density; (**c**) is the evolution of aspect ratio of the voids.

Figure 7 shows the quantification of the damage evolution in the necking zone. The increase in porosity (volume fraction of voids) is similar for all the microstructures at the thickness strain below 0.15, and it seems that the damage starts to accumulate right after the onset of necking. At large strains, the porosity of Equiaxed-49% and Fibrous-26% are similar, which is higher than the sample of Fibrous-45%. Differences could also be observed in terms of the evolution of void density. Fibrous-26% involves the enhanced formation of voids, while Fibrous-45% present the lowest potency of void nucleation. In all these microstructures, the nucleation of voids is continuous during the entire deformation process. Therefore, Fibrous-45% exhibits the highest resistance to damage accumulation, especially comparing withEquiaxed-49% that involves a similar amount of martensite. The formation of elongated voids is also justified by the aspect ratio from 1.6 to 2.2 (Figure 7c).

**Figure 8:**
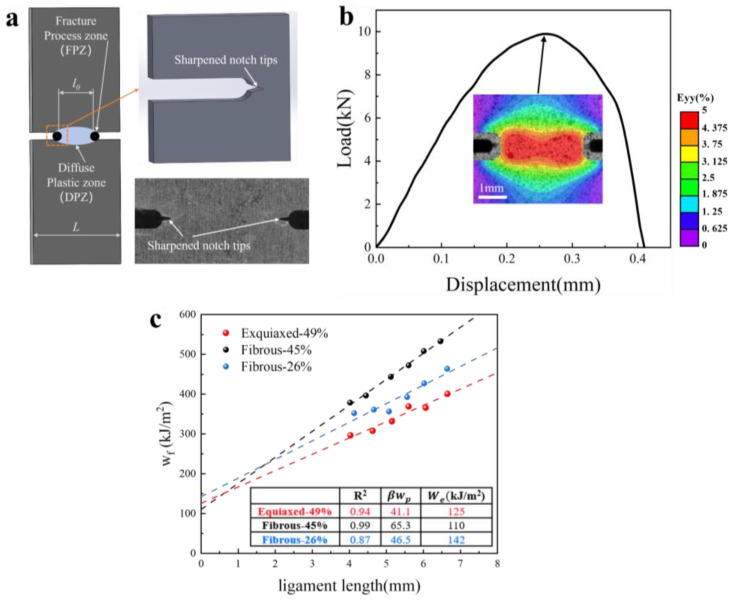
Fracture resistance evaluated by the EWF method: (**a**) is the design of the DENT specimen; (**b**) is the typical load-displacement curve and the evolution of notch opening and strain field; (**c**) is the data of the EWF measurements.

The fracture resistance was further assessed with the EWF method. Figure 8 shows the design of the DENT specimens for the measurement of EWF associated with the sharpened notch tips. During the DENT tension, the notch tips were significantly blunted, and the crack extension was observed after a substantial plastic deformation of the ligament, which was revealed by the strain measurement using digital image correlation. The deformation process of the DENT specimens justifies the application of the EWF method [30]. The measurements of *w_f_* with various ligament lengths (*l*_0_) is summarized in Figure 8c. It is shown that *w_f_* varies approximately linearly with *l*_0_ for the microstructures under investigation, which substantiates the good quality of the experimental campaign. Fibrous-45% shows a higher level of *w_f_* than Equiaxed-49%, but the linear regression results in a similar value of *w_e_*. Fibrous-26% generally shows a slightly higher value of *w_f_* than Equiaxed-49%, but the value of *w_e_* is the highest among the microstructures under investigation. Therefore, when assessing the fracture properties by using the EWF method, Fibrous-26% presents the best fracture resistance, and Fibrous-45% and Equiaxed-49% demonstrate a similar performance.

**Figure 9:**
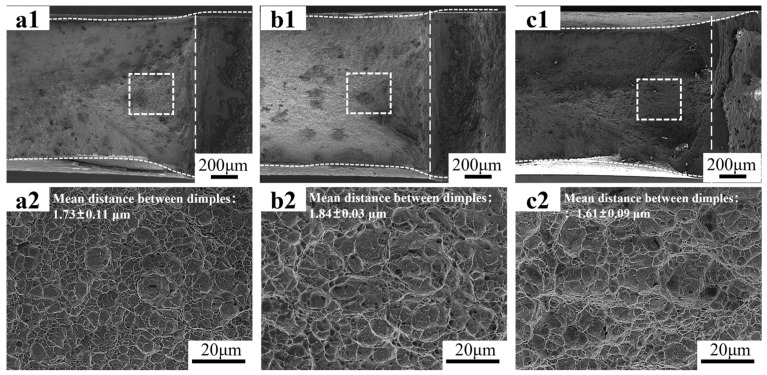
Fracture surface analysis of the broken DENT specimens: (**a1**,**a2**) are the SEM micrographs of Equiaxed-49%; (**b1**,**b2**) are the SEM micrographs of Fibrous-45%; (**c1**,**c2**) are the SEM micrographs of Fibrous-26%.

Figure 9 shows the fracture surface of the broken DENT specimens. All the DENT specimens present the flat fracture at crack initiation, and then transit to the slant fracture, which is typical of the cracking of sheet metals with a high strength [35]. The fracture surfaces are covered by micro-sized dimples with equiaxed shape. The mean distance between dimple centers was measured, and it is surprising that such values are similar among the microstructures and are also similar to that under uniaxial tension. Fibrous-26% involves a higher equivalent fracture strain ($\varepsilon_{f}^{eq}=$ 0.40) in the DENT tension, when compared with Fibrous-45% ($\varepsilon_{f}^{eq}=$ 0.20) and Equiaxed-49% ($\varepsilon_{f}^{eq}=$ 0.29).

**Figure 10:**
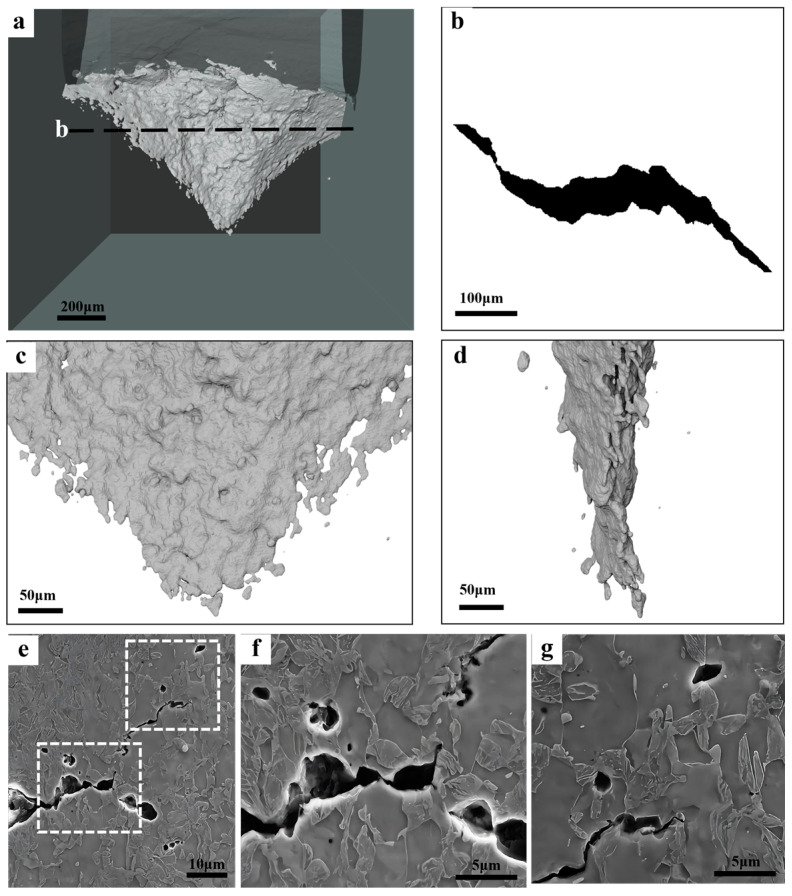
Characterization of the cracking behavior in the DENT specimen of Equiaxed-49%: (**a**) is the overview of the crack; (**b**) is the crack profile along the thickness direction as indicated in (**a**); (**c**,**d**) are the magnified micrographs of the crack tip obtained from different directions; (**e**–**g**) are the SEM micrographs of the damage events at the crack tip, with (**f**,**g**) the high-magnitude micrographs of the areas indicated in (**e**).

Interrupted tests were performed on the DENT specimens, and a combination of X-ray CT and SEM was used to reveal the cracking behavior. Figure 10 shows the observations on the sample of Equiaxed-49%. The very beginning of cracking initiation was successfully captured, which is located at the center of the sheet due to the highest stress triaxiality. Such crack tunneling effect could significantly influence the detection of crack initiation and could result in an overestimation of fracture initiation toughness based on the observation of surface cracking [5,25]. Direct observation has shown that the crack extends in a discontinuous way by joining the micro-sized voids adjacent to the crack tip. The profile of the crack shows that the flat fracture only dominates at the very early stage of cracking, and the flat-to-slant transition occurs immediately after the crack initiation. Furthermore, the slant fracture also occurs in a discontinuous manner. The observations on the mid-thickness section show that the voids in the near-crack tip region are formed by martensite cracking. Significant void coalescence occurs in the near-crack tip region, which results in the crack extension.

**Figure 11:**
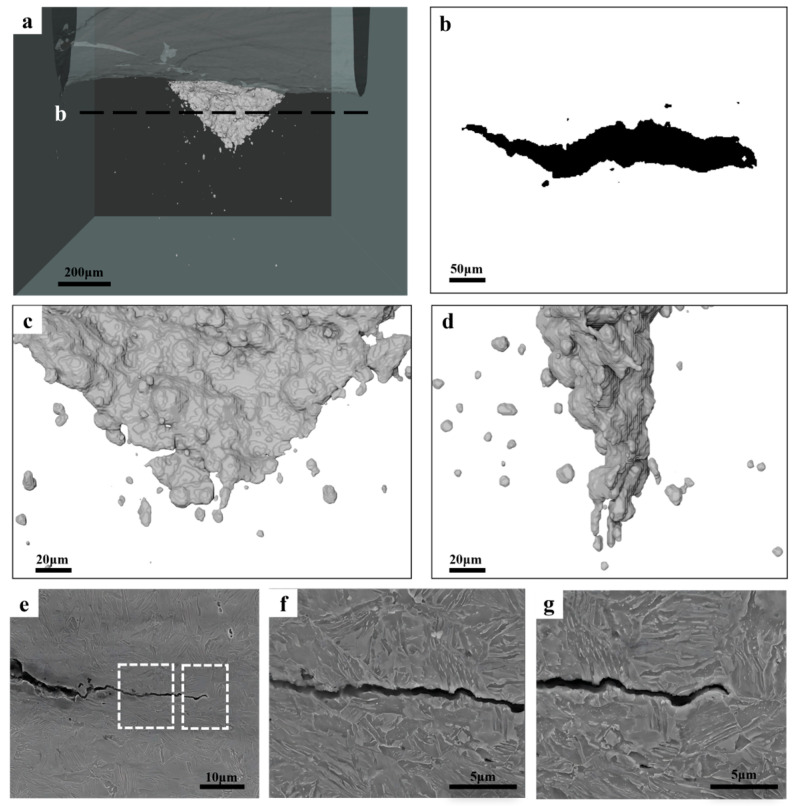
Characterization of the cracking behavior in the DENT specimen of Fibrous-45%: (**a**) is the overview of the crack; (**b**) is the crack profile along the thickness direction as indicated in (**a**); (**c**,**d**) are the magnified micrographs of the crack tip obtained from different directions; (**e**–**g**) are the SEM micrographs of the damage events at the crack tip, with (**f**,**g**) the high-magnitude micrographs of the areas indicated in (**e**).

**Figure 12:**
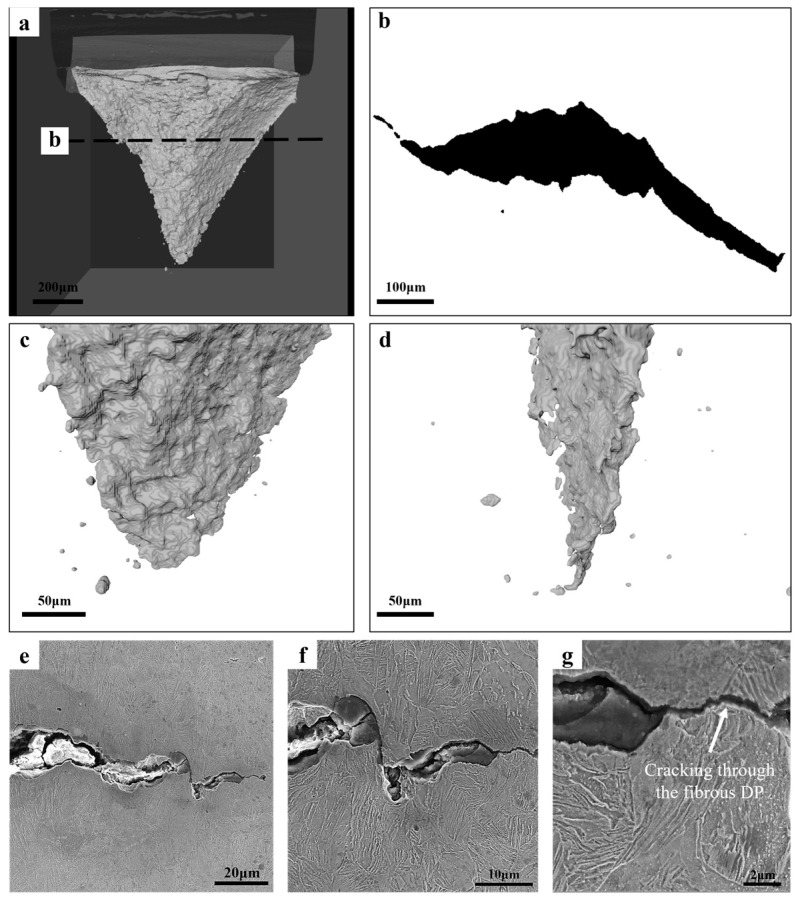
Characterization of the cracking behavior in the DENT specimen of Fibrous-26%: (**a**) is the overview of the crack; (**b**) is the crack profile along the thickness direction as indicated in (**a**); (**c**,**d**) are the magnified micrographs of the crack tip obtained from different directions; (**e**–**g**) are the SEM micrographs of the damage events at the crack tip.

Figure 11 shows the cracking behavior of the DENT specimen of Fibrous-45%. It shows a similar crack morphology with that in Figure 10, i.e., the cracking initiation at the center of the sheet associating with crack tunneling effect. The flat-to-slant transition occurs right after the crack initiation, and it is observed that the slant fracture is also proceeded by the joining the voids adjacent to the crack tip. Observations have been made for the crack extension during slant fracture. More localized damage process is involved, in that micro-size voids are only along the crack plane, without the void distribution around the near-crack tip region as shown in Figure 10. The characterization of the cracking behavior has also been made for Fibrous-26%, and the results are provided in Figure 12. It can be seen that Fibours-26% is also presenting a significant crack tunneling effect, associated with the flat-to-slant transition. In addition, according to the SEM micrographs, the crack is reflected during the propagation, involving the pathway going through the fibrous domain.

## 4. Discussion

The morphology of the ferrite–martensite dual-phase microstructure depends on the processing history, presenting a remarkable space for microstructure optimization. In the production of the conventional DP steels, the cold-rolled ferrite–pearlite microstructure is usually chosen as the initial microstructure, which is subjected to the intercritical annealing to form the ferrite–austenite microstructure and then quenched to room temperature for the ferrite–martensite mixture [4]. Therefore, the features of the cold-rolled DP microstructure are controlled by the interplay between ferrite recrystallization and austenite formation. The austenite tends to form at the carbide on the ferrite grain boundaries, and it is the degree of ferrite recrystallization that determines both the ferrite grain size and the distribution of martensite islands [36]. The ferrite recrystallization is fast at high temperatures, and the achievement of ultrafine-grained DP steels is challenging, requiring the techniques of, e.g., ultrafast heating [37] and severe plastic deformation [15]. In this sense, the fibrous DP concept is an elegant way of exploring the grain refinement effect, in that only simple heat treatment is needed. Instead of using the cold-rolled ferrite–pearlite microstructure, the lath martensite is designed as the initial microstructure for the generation of fibrous DP steels. During the heating and soaking of intercritial annealing, the austenite could nucleate at the prior austenite grain boundaries and at the lath boundaries, which are enriched with carbon due to the segregation effect [38,39] or due to the pre-existence of retained austenite [40,41]. In this study, the martensite formed at the prior austenite grain boundaries is of the granular shape, and that formed from the lath boundaries is of the plate-like shape. The aggregate of the plate-like martensite islands is the key ingredient of the fibrous DP morphology. However, as the volume fraction of martensite is increased, the proportion of the granular martensite islands increases and the plate-like martensite becomes to be impinged, which tends to reduce the characteristics of the fibrous morphology.

The results of tensile testing clearly show the efficiency of the fibrous morphology in strengthening the materials, which is consistent with the results in [21]. Although with a much lower martensite volume fraction, Fibrous-26% presents a higher yield strength than Equiaxed-49%. The strengthening efficiency of the fibrous DP steels originates primarily from the ferrite grain refinement. In the lath martensite, the lath size ranges from 30 nm to 1 μm, with the average value of 200 nm [39]. The lath boundaries involve a small misorientation between the neighboring grains, and the lath size is, thus, not the effective structural size for martensite [42,43]. However, after the intercritial annealing, the austenite reversion and the subsequent formation of the plate-like martensite has changed the lath boundaries into the high-angle grain boundaries, effectively reducing the ferrite grain size. According to the statistical measurement, the average ferrite grain size in Fibrous-26% and Fibrous-45% are 0.8 μm and 0.9 μm, respectively, while the average ferrite grain size in Equiaxed-49% is 4.0 μm. However, the ferrite grain refinement is also decreasing the strain hardening capability due to the insufficient dislocation storage [44]. As shown in Figure 4, the uniform elongation of Fibrous-26% and Fibrous-45% are far lower than Equiaxed-49%.

A previous study [19] reported that the fibrous DP steels of the strength of ~800 MPa possess a high fracture resistance both under uniaxial tension and in the EWF measurements, which was primarily attributed to the delayed damage nucleation. In this study, the fibrous morphology has more complicated influences on the fracture properties. Fibrous-26% demonstrates the best fracture properties both under uniaxial tension and DENT tension. However, in Fibrous-26%, the damage nucleation is most significant among the microstructures under investigation, which could be attributed to the higher carbon content in martensite and, thus, to the higher hardness. A higher strength contrast between phases tends to promote the occurrence of interface decohesion [45]. For Fibrous-45%, the damage accumulation is slower than Equiaxed-49%. Consistent with [19], the major mechanism of damage nucleation in Fibrous-45% is interface decohesion, in contrast to the martensite cracking mechanism in Equiaxed-49%. The dominance of interface decohesion in Fibrous-45%, which involves a high martensite volume fraction, should be related to the small size of martensite islands and, thus, the reduced probability to nucleate an internal microcrack in martensite.

When the fracture resistance is assessed with the EWF method, which involves a higher stress triaxiality and stress concentration, a different ranking is the result (Figure 8c). The essential work of fracture is evaluating the resistance for crack propagation [5,32]. The X-ray CT of the interrupted tests on all the specimens show the transition from flat fracture to slant fracture at the very beginning (Figure 10, Figure 11 and Figure 12), which is typical of the sheet metals of high strength. Fibrous-26% involves the highest value of *w_e_*, while the *w_e_* of Fibrous-45% and Equiaxed-49% are similar. For the ductile thin sheets, the essential work of fracture includes two energy contributions, including the intrinsic work spent for damage and fracture (*Г*_0_) and an average work spent in crack tip necking (*Г_n_*) [19,46,47]. The intrinsic work of *Г*_0_ scales with the product of strength and spacing of voids [48], which could be approximated to the mean spacing between dimple centers. According to the quantification of dimples in Figure 9, Fibrous-26%, Fibrous-45%, and Equiaxed-49% should have similar magnitude of *Г*_0_. On the other hand, as shown in Figure 9, Fibrous-26% presents the highest equivalent fracture strain, which suggests a larger contribution to the work for crack tip necking *Г_n_*, and, thus, explains the higher value of *w_e_*. Therefore, the fibrous morphology is efficient in increasing the yield strength of Fibrous-26% due to the ferrite grain refinement effect, and the associated larger crack tip necking contributes to a higher fracture toughness, resulting in the optimized combination of strength and fracture resistance.

## 5. Conclusions

In this study, the mechanical properties of DP steels with equiaxed and fibrous morphologies were investigated systematically with the testing configurations of uniaxial tension and DENT tension. The major findings are listed as follows:

(1) The fibrous morphology is efficient in increasing strength due to the ferrite grain refinement effect, but the uniform elongation is compromised by a lower strain hardening capability.
(2) The DP microstructures of Equiaxed-49%, Fibrous-45%, and Fibrous-26% involve a tensile strength above 1 GPa, but the damage mechanism is different. The major damage mechanism is martensite cracking in Equiaxed-49%, while that is interface decohesion in the fibrous microstructures.
(3) Under uniaxial tension, the fibrous morphology does not improve the fracture strain. But under DENT tension, the measurements of EWF show that Fibrous-45% and Equiaxed-49% have similar fracture resistance, and the Fibrous-26% presents the highest value of *w_e_*. The superior *w_e_* of Fibrous-26% is presumably attributed to the larger energy dissipation by crack tip necking.

## Figures and Tables

**Table 1:** Tensile properties of the microstructures under investigation.

	YS (MPa)	UTS (MPa)	UE	Area Reduction	Fracture Strain	Fracture Stress (MPa)
Equiaxed-49%	707 ± 7	1005 ± 11	0.104 ± 0.004	0.72 ± 0.05	1.31 ± 0.08	3711 ± 32
Fibrous-45%	837 ± 12	1052 ± 8	0.058 ± 0.013	0.66 ± 0.03	1.08 ± 0.02	3064 ± 59
Fibrous-26%	779 ± 9	1004 ± 14	0.061 ± 0.018	0.73 ± 0.07	1.30 ± 0.05	3692 ± 44

YS, yield strength; UTS, ultimate tensile strength; UE, uniform elongation.

## Data Availability

The original contributions presented in this study are included in the article. Further inquiries can be directed to the corresponding authors.
